# A novel microwave dielectric ceramic Li_2_NiZrO_4_ with rock salt structure

**DOI:** 10.1039/c9ra07308f

**Published:** 2019-10-15

**Authors:** Pengbo Jiang, Yongda Hu, Shengxiang Bao, Jie Chen, Zongzhi Duan, Tao Hong, ChengHao Wu, Gang Wang

**Affiliations:** University of Electronic Science and Technology of China Chengdu 610054 China 3097213743@qq.com; Chengdu Yaguang Electronics Co. Ltd., Microwave Circuit & System Institute Chengdu 610054 China

## Abstract

Low loss Li_2_NiZrO_4_ ceramics with rock salt structure were successfully prepared by the solid-phase reaction method. The relationship between sintering temperature, phase composition and dielectric properties of Li_2_NiZrO_4_ ceramics was reported for the first time. The grain size gradually increased and the porosity decreased with the sintering temperature increasing. When the sintering temperature exceeds 1300 °C, the grains grow abnormally and some grains begin to melt. The XRD patterns indicated the second phase ZrO_2_ appeared due to the volatilization of lithium. The grains grow abnormally and a second phase of ZrO_2_ increased the loss of Li_2_NiZrO_4_ ceramics. The samples sintered at 1300 °C possessed the best dielectric properties: *ε*_r_ = 12.3, *Q*_f_ = 20000 GHz, *τ*_f_ = −23.4 ppm °C^−1^, which would make the ceramic a possible candidate for millimeter-wave applications.

## Abstract 2

Low loss Li_2_NiZrO_4_ ceramics with rock salt structure were successfully prepared by the solid-phase reaction method.

## Graphical abstract

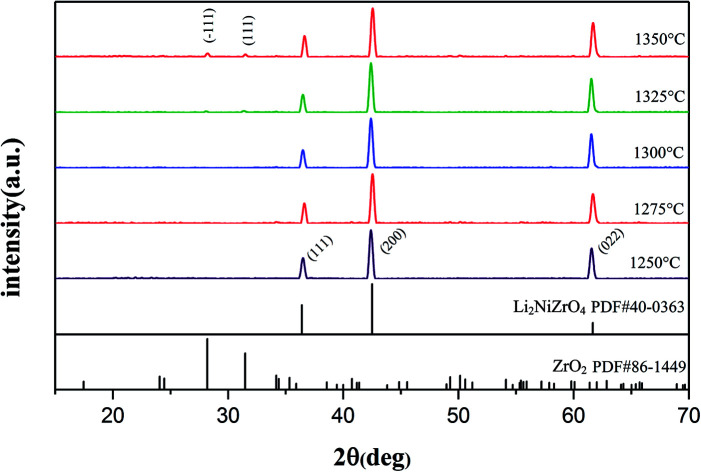

## 1. Introduction

Due to the rapid development of wireless communication systems, dielectric ceramics have been widely investigated as resonators, dielectric substrates and dielectric waveguide circuits in contemporary communications. It performs a significant function in millimeter wave communication as the substrate materials of microwave integrated circuits.^1^ To satisfy the demand of the fast-paced expansion of communications, these materials need to have a suitable dielectric constant to meet miniaturization of the device. In order to reduce the loss of the device at high frequencies, the quality factor is also required to be as large as possible.^2^ At the same time, in order to ensure temperature stability, a temperature coefficient of the resonance frequency close to zero is also required.^3^

In recent years, it has been extensively indicated that the mixed Li_2_O-AO-BO_2_ (A = Mg, Zn and Ni; B = Ti, Zr and Sn) system is quite appropriate for microwave communication. Out of these microwave dielectric ceramics, The Li-containing Li_2_MgTiO_4_ ceramic is adopted as a perfect microwave dielectric material, which is an appropriate candidate for the component miniaturization and integration.^4–6^ Li_2_MgTiO_4_ with the microwave dielectric characteristics of *ε*_r_ = 15.07, *Q*_f_ = 97629 GHz (at 8.2 GHz) and *τ*_f_ = 3.81 ppm °C^−1^ was reported by Pan *et al.*^7^ Additionally, Zhang *et al.* investigated the phase composition of (1 − *x*)Li_2_TiO_3_-*x*NiO (0 ≤ *x* ≤ 0.5) ceramics and obtained excellent microwave dielectric characteristics: *ε*_r_ = 19, *Q*_f_ = 62252 GHz and *τ*_f_ = −1.65 ppm °C^−1^ for *x* = 0.2.^8^ The Li_2_ZrO_3_-AO ceramic system was investigated. Ma *et al.* indicated the impact of ZnO addition on the microwave dielectric characteristics of Li_2_ZrO_3_ ceramics and obtained microwave dielectric characteristics of 0.7Li_2_ZrO_3_-0.3ZnO ceramics: *ε*_r_ = 14.8, *Q*_f_ = 26800 GHz and *τ*_f_ = 1 ppm °C^−1^.^9^ Bi *et al.* reported the microwave dielectric characteristics of Li_2_MgZrO_4_ ceramics: *ε*_r_ = 12.30, *Q*_f_ = 40900 GHz, besides *τ*_f_ = −12.31 ppm °C^−1^ when it was sintered at 1175 °C for 4 h.^10^ Cheruku *et al.* synthesized Li_2_NiZrO_4_ materials with LiNO_3_, Ni(NO_3_)_2_·6H_2_O, ZrN_2_O_7_ and C_6_H_6_O_7_ by solution combustion technique in phase pure nanocrystalline form for the first time. They found the electrical relaxation is essentially non-Debye and temperature independent. This material exhibits considerable conductivity at room temperature and is a possible candidate for electrode material in solid-state batteries.^11,12^ Nevertheless, there have no report about the microwave dielectric characteristics of Li_2_NiZrO_4_ materials. In the present work, the sintering temperature, density as well as microwave dielectric properties of Li_2_NiZrO_4_ ceramics were investigated. Besides, the relationship existing between phase composition, sintering temperature, microstructure and microwave dielectric characteristics of Li_2_NiZrO_4_ ceramic was also investigated.

## 2. Experiment

The starting materials Li_2_CO_3_(99.99%, Aladdin), ZrO_2_(99.9%, Aladdin) and NiO (99.99%, Aladdin) were used to fabricate Li_2_NiZrO_4_ ceramic by solid state reaction methodology. First, the prepared powder is put into a nylon can and ball milled for 5 h with alcohol as the medium. Thereafter, the powder was dried and pre-sintered at 1050 °C for 4 hours. The calcined powder milling was carried out again for 7 h and followed by drying. In addition, the dried powder was mixed with polyvinyl alcohol for the purpose of forming a pellet. The pellets were pressed into a cylinder shape (diameter: 12 mm, thickness: 6 mm) under the pressure of 10 MPa. Eventually, the specimens were muffled with the same material powder and sintered at 1250–1350 °C for 5 h.

The measurement of the density of the dielectric ceramic was carried out with the help of the Archimedes method. In addition, the testing for the phase formation was carried out by X-ray diffractometer (XRD) (Tongda TDM-20) with Cu-Kα radiation. The microstructure of the specimen was observed by scanning electron microscope (SEM) (AURA-100, Seron, South Korea). The measurement of the dielectric constant (*ε*_r_) as well as quality factor (*Q*_f_) was carried out by network analyzer (E5061B, KEYSIGHT) on the basis of the Hakki–Coleman dielectric resonator methodology.^13–15^ The calculation of the temperature coefficient of resonant frequency (*τ*_f_) is performed in accordance with the formula: 
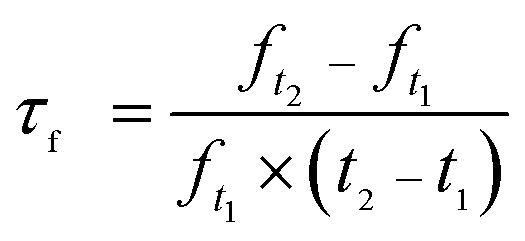
where *f*_*t*_1__ and *f*_*t*_2__ are the resonant frequencies at the temperature *t*_1_ = 25 °C and *t*_2_ = 85 °C, correspondingly.

## 3. Results and discussion

**Fig. 1:**
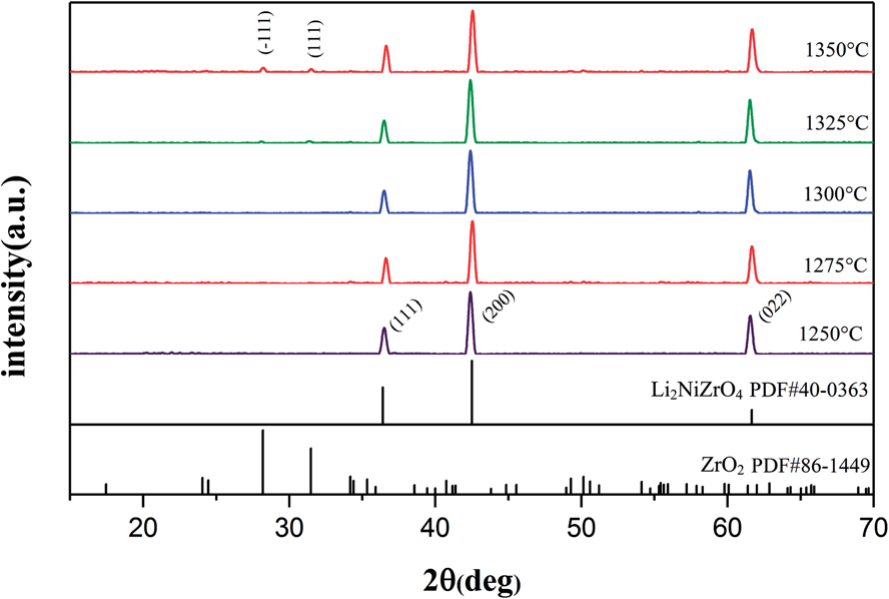
XRD patterns of the Li_2_NiZrO_4_ sintered at 1250–1350 °C.

The XRD patterns of Li_2_NiZrO_4_ ceramics sintered between 1250–1350 °C are presented in Fig. 1. As seen, pure phase Li_2_NiZrO_4_ (PDF#40-0363) ceramics with rock salt structure was formed. When the sintering temperature reached 1325–1350 °C, the presence of the ZrO_2_ (PDF#86-1449) secondary phase is observed.^16^ Y. Iida *et al.* indicated the fact that the volatilization of lithium is obvious upon sintering at 1000 °C.^17^ This situation was also observed in LiMO_3_ (M = Nb, Ta) crystals.^18–20^ Therefore, it is taken into account that the volatilization of lithium gives rise to the formation of ZrO_2_ with the sintering temperature exceeding 1300 °C.^10^

**Fig. 2:**
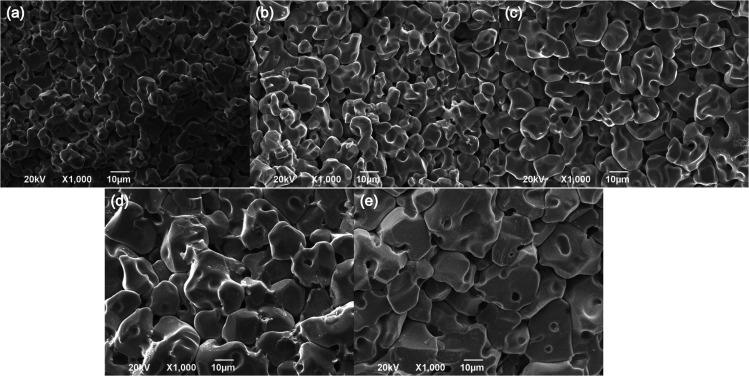
Fracture SEM images of Li_2_NiZrO_4_ ceramics with different sintering temperatures for 5 h (a–e corresponds to 1250 °C, 1275 °C, 1300 °C, 1325 °C and 1350 °C).

The SEM micrographs of Li_2_NiZrO_4_ sample sintered at different temperatures for 5 h are shown in Fig. 2. It is seen that the grain size of Li_2_NiZrO_4_ ceramics gradually increases as the sintering temperature is higher. A number of intergranular pores can be observed in the Fig. 2(a) and (b).With the sintering temperature growth, the grain size increased substantially and few pores are observed in Fig. 2(c). These pores are caused by lithium volatilizing. As the sintering temperature ranged from 1325 to 1350 °C, the grains showed abnormal growth. Moreover, some grains start melting, and a small amount of pores is still existed owing to the lithium volatilization.^10,21,22^

**Fig. 3:**
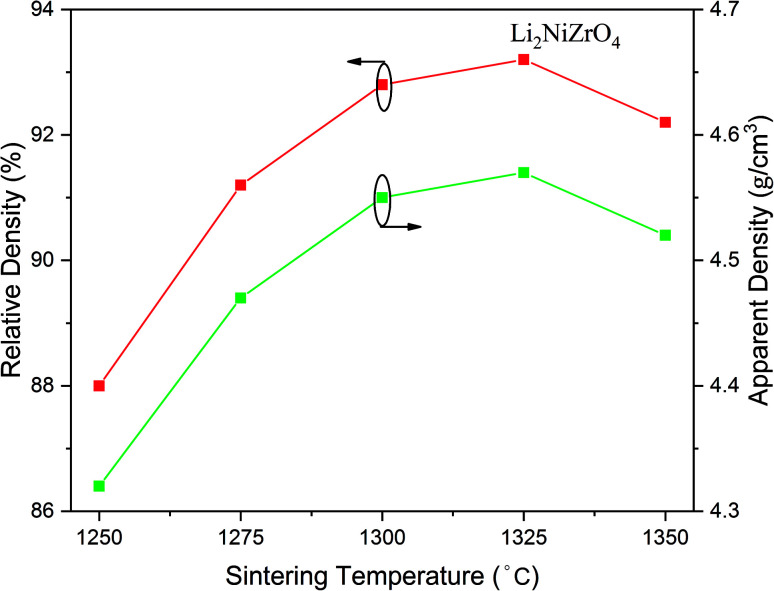
Apparent density and relative density of Li_2_NiZrO_4_ ceramics sintered at different temperatures for 5 h.

Fig. 3 presents the variation of the apparent density as well as relative density of Li_2_NiZrO_4_ ceramics. With the sintering temperature increase from 1250 to 1325 °C, the apparent density increased from 4.32 to 4.57 g cm^−3^. When the sintering temperature was 1350 °C, the apparent density was 4.52 g cm^−3^. The theoretical density of Li_2_NiZrO_4_ crystal is 4.9 g cm^−3^. As the sintering temperature amounted to 1300 °C, the relative density was 92.8%. As the sintering temperature becomes higher, there is a gradual growth of the grain size, together with the pore declining, which is in appropriate relation to the variation in density.

**Fig. 4:**
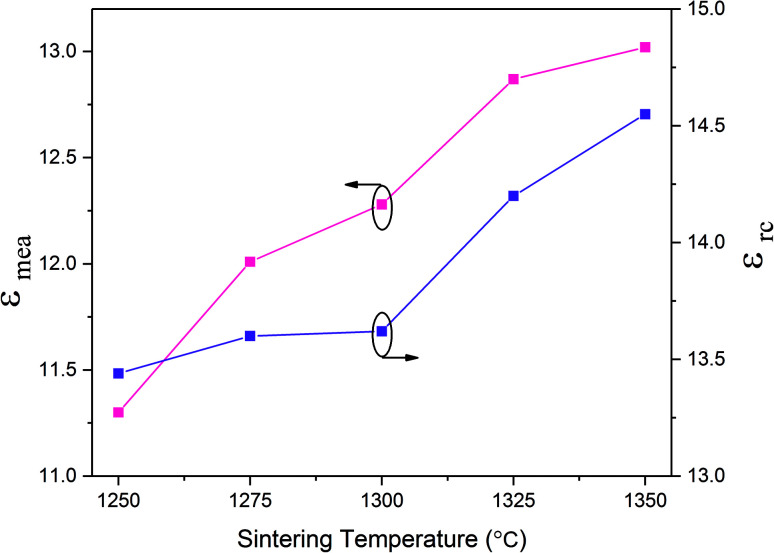
Measured permittivity (*ε*_mea_) and porosity-corrected permittivity (*ε*_rc_) of Li_2_NiZrO_4_ ceramics sintered at different temperatures for 5 h.

The variation in dielectric constant of Li_2_NiZrO_4_ ceramics as a function of sintering temperature is given in Fig. 4. With the sintering temperature increasing from 1250 to 1300 °C, the *ε*_r_ value continuously increased. The variation in *ε*_r_ value was consistent with that in the apparent density. Ordinarily, many factors affect the dielectric constant, such as ionic polarizability, density and second phase.^23^ In the present work, the dielectric constant increases with increasing sintering temperature, which is related to relative density, polarizability and the second phase. The relationship between relative permittivity, polarizability and relative density can be described by Clausius–Mosotti equation (see eqn (1)).^24^1
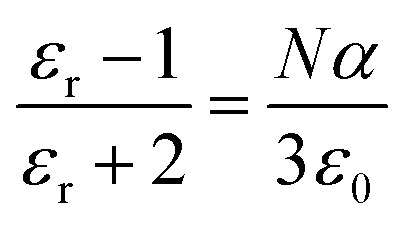
where *α*, *ε*_0_, and *ε*_r_ are the molecular polarizability, permittivity of the vacuum and dielectric ceramics, respectively. The theoretical dielectric polarizability (*α*_theo_) of Li_2_NiZrO_4_ ceramics can be obtained according to the Shannon's additive rule (see eqn (2)).^25^

2 *α*_theo_ = 2*α* (Li^+^) + *α* (Ni^2+^) + *α* (Zr^4+^) + 4*α* (O^2−^)

Where *α* (Li^+^) = 1.20 Å^3^, α (Ni^2+^) = 1.23 Å^3^, α (Zr^4+^) = 3.25 Å^3^ and *α* (O^2−^) = 2.01 Å^3^ are the ionic polarizabilities. And as the porosity fraction increases, the dielectric constant will decrease, seen from eqn (1). In order to eliminate the influence of porosity on dielectric, dielectric is corrected by eqn (3).^26,27^3
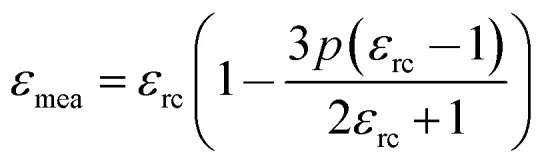
where *p* is porosity the fraction, *ε*_mea_ is measured permittivity and *ε*_rc_ is the porosity-corrected permittivity. The observed dielectric polarizability (*α*_obs_) is calculated using eqn (2) (see eqn (4)).^28^4
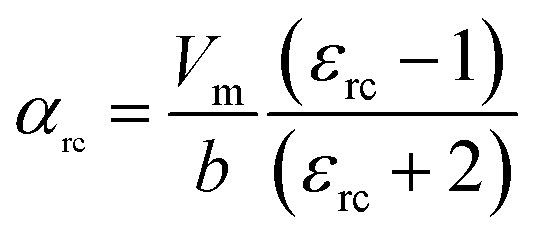
where *b* and *V*_m_ are the constant (4π/3) and the molar volume, respectively. According to eqn (1), The theoretical dielectric polarizability (*α*_theo_) of Li_2_NiZrO_4_ ceramics is 14.92. The observed dielectric polarizability (*α*_obs_) is 14.88 by eqn (2) and (3) (sintered at 1300 °C). Obviously, there is a very small difference of (*Δ* = |(*α*_theo_ − *α*_rc_)/*α*_rc_ × 100%|) ≤1%, indicating that the values of *α*_theo_ and *α*_obs_ are basically the same. As the sintering temperature continues to increase, the relative density decreases and the dielectric constant continues to increase. This phenomenon indicates that the dielectric constant is also affected by the second phase. When the sintering temperature reaching 1350 °C, the ZrO_2_ second phase is formed and possess a higher dielectric constant value of 23.^29^ So the relative dielectric constant continues to increase while the relative density decreases.^30,31^

**Fig. 5:**
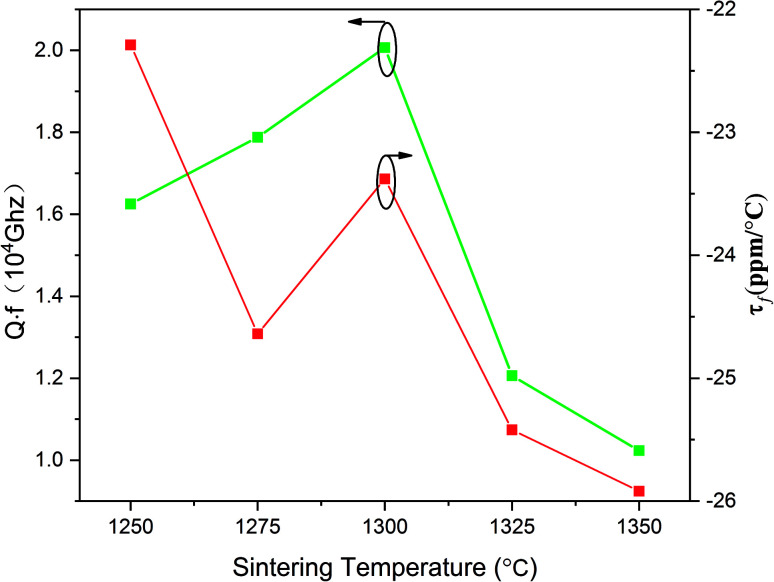
Quality factor (*Q*_f_) and temperature coefficient of the resonant frequency (*τ*_f_) of Li_2_NiZrO_4_ ceramics sintered at different temperatures for 5 h.

In Fig. 5, the variations of the quality factor and temperature coefficient of the resonant frequency are presented. There is an increase in the *Q*_f_ value from 16 000 to 20 000 GHz with the sintering temperature increasing from 1250 to 1300 °C. The *Q*_f_ value reached the topmost value of 20 000 at 1300 °C. Nonetheless, the *Q*_f_ value sharply declined with the sintering temperature ranging from 1325 to 1350 °C and the *Q*_f_ value is merely 10 200 GHz at 1350 °C. In general, dielectric loss can be divided into two categories. One component is a result of the bulk crystal phase, termed as intrinsic dielectric loss while the other component is termed as extrinsic dielectric loss, such as the grain boundaries, flaws and so on. With the sintering temperature increasing from 1250 to 1300 °C, there is an increase in the grain size as well as the apparent density, which shares a similarity with the variation of *Q*_f_ value. When the sintering temperature kept rising, the abnormal grain growth and second phase precipitation led to the decline of *Q*_f_ value.^32^ The trend of *τ*_f_ with the sintering temperature is also shown in Fig. 5. With the sintering temperature ranging from 1250 and 1350 °C, the *τ*_f_ value fluctuates between −22.29 and −25.92 ppm °C^−1^.

## 4. Conclusion

The Li_2_NiZrO_4_ ceramic was successfully prepared by solid-state reaction method. The phase composition, microstructure and microwave dielectric properties of Li_2_NiZrO_4_ ceramics were studied. With the sintering temperature exceeding 1300 °C, the ZrO_2_ second phase was formed owing to the lithium volatilization. The *ε*_r_ is dependent on second phases and density. The *ε*_r_ gradually increases as the density increases with the sintering temperature ranging 1250–1325 °C. When the sintering temperature reaching 1350 °C, the ZrO_2_ second phase possess a significant effect on the *ε*_r_. The *Q*_f_ value was primarily constrained by the microstructure and second phase. The samples sintered at 1300 °C showed the optimal dielectric characteristics: *ε*_r_ = 12.3, *Q*_f_ = 20 000 GHz, *τ*_f_ = −23.38 ppm °C^−1^ that made the ceramic promising for millimeter-wave applications.

## Conflicts of interest

There are no conflicts to declare.

## Supplementary Material
